# Incorporating Transition Metal Ions into Uranium Oxide Hydrates: The Role of Zn(II) and the Effect of the Addition of Cs(I) Ions

**DOI:** 10.1021/acsomega.4c06188

**Published:** 2024-08-23

**Authors:** Timothy A. Ablott, Kimbal T. Lu, Yingjie Zhang

**Affiliations:** Australian Nuclear Science and Technology Organisation, Locked Bag 2001, Kirrawee, New South Wales 2232, Australia

## Abstract

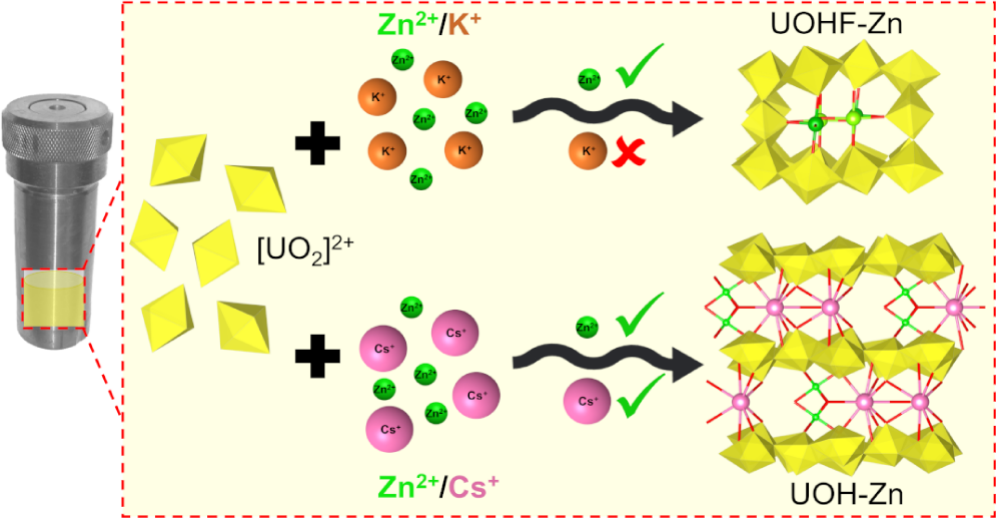

The synthesis of two zinc-bearing uranium oxide hydrate (UOH) materials has been achieved, and their crystal structures, obtained via single-crystal X-ray diffraction using synchrotron radiation, and additional structural and spectroscopic properties are reported herein. Although both structures incorporate Zn^2+^ cations, the two differ significantly. The compound Zn_2_(OH)_2_(H_2_O)_5_[(UO_2_)_10_UO_14_(H_2_O)_3_] (**UOHF-Zn**), forming a framework-type structure in the *P*1̅ space group, was composed of β-U_3_O_8_ layers pillared by uranyl polyhedra, with the Zn^2+^ cations incorporated within the framework channels. In contrast, the compound Cs_2_Zn(H_2_O)_4_[(UO_2_)_4_O_3_(OH)_4_]_2_·3H_2_O (**UOH-Zn**) crystallized in the *Cmc*2_1_ space group with a schoepite-like uranyl oxide hydroxide layered topology and both Zn^2+^ and Cs^+^ cations making up the interlayer species. The apparent driving force for the differences in the structures was the change from KOH to CsOH during synthesis, with the smaller K^+^ ions excluded in lieu of a higher proportion of Zn^2+^ (U/Zn ratio of 5.5:1) in **UOHF-Zn**, whereas in **UOH-Zn**, the larger Cs^+^ ions were preferentially incorporated at the expense of fewer Zn^2+^ cations (U/Cs/Zn ratio of 8:2:1). Highlighted in this work is the effect of the chemical species and, in particular, their ionic radius on UOH formation, further improving the understanding of UO_2_ alteration in the setting of deep geological repositories.

## 1 Introduction

The international drive toward combating climate change has seen nuclear energy gain renewed interest in recent years.^1^ However, ever-present in the discussion around uranium oxide (UO_2_) based nuclear fuel systems is the consideration of how to safely deal with the spent nuclear fuel (SNF) toward keeping both the general public and the environment safe in the centuries to follow.^2,3^ As these considerations progress, many countries are looking to the storage of their SNF in deep, stable geological repositories.^4−6^

With UO_2_ being the primary uranium species present in these repositories and the understanding that, if exposed to air or water, these materials can readily proceed along oxidation and hydration alteration pathways,^7,8^ a major focus has been on further elucidating the chemistry of these systems. The most prevalent of these studies has been on the alteration of the mineral uraninite (UO_2+*x*_), which is known to undergo oxidation from U^4+^ to U^6+^ under oxidative conditions.^9,10^ The resultant uranyl species, [UO_2_^2+^], are highly reactive and readily undergo interactions with their surroundings to form a plethora of uranyl oxide-based compounds.^11,12^ These materials are typically composed of strongly bound axial oxygen atoms, as part of the uranyl bond of the UO_2_^2+^ cation, and coordinated ligands that extend along the equatorial plane via bound O/OH groups. The result is typical uranium coordination geometries of tetragonal, pentagonal, and hexagonal bipyramids.^13,14^ These uranium polyhedra extend through edge- and corner-sharing and often result in layered structures, wherein any additional metals taken up from their surroundings are incorporated as a secondary layer, which lies between these uranium sheets.^15,16^

One such subset of these materials are uranium oxide hydrate (UOH) compounds, which have been identified as forming during the early stages of the alteration of uraninite.^12^ Given this, they have been proposed as a means of studying the alteration of UO_2_-based SNF systems in geological repositories.^17^ This focus has resulted in the identification of dozens of UOH-related mineral structures,^11,12,18^ along with a growing library of synthetically produced UOH compounds.^15,19,20^ The commonalities in these systems include recurring uranium oxide hydroxide sheet-like topologies, between which is found the interlayer cation species. The differentiating factors in these materials are 2-fold: the identity of the interlayer cations and the O/OH ratio of the uranium oxide hydroxide layer. In examining the identified interlayer cations, some trends are apparent. In naturally occurring UOH systems, the incorporated cations have been found to be mostly alkali^21,22^ and alkaline earth^23,24^ species, along with a prevalence of Pb-based minerals, expected given its position at the end of the U decay pathway.^25,26^ Notably lacking are UOH minerals containing transition metals, despite their relative abundance in the Earth’s crust. A means of filling the gaps in these UOH systems has thus been the generation of a range of synthetic UOH compounds, with alkali metals,^21^ alkaline earth metals,^27^ transition metals,^28^ heavy metals,^29^ and lanthanides^30^ all identified as the secondary cation incorporated into these structures. Thus, this has proven to be an effective method to broaden the scope of possible UOH compounds as the alteration pathway of UO_2_-based materials.

Upon examining these systems, what was also identified was a more complex substructure of the typical layered UOH structure, wherein the axial oxygens are also coordinated, acting as bridging ligands between the uranyl polyhedra layers to form framework-type structures, since termed uranium oxide hydrate frameworks (UOHFs). These frameworks give rise to channels that extend throughout the structure, and it is into these channels that the secondary cations are incorporated, with compounds containing Cs^+^, Sr^2+^, Cd^2+^, Pb^2+^, Y^3+^, Er^3+^, Sm^3+^, Eu^3+^, Gd^3+^, and U^4+^ all identified.^27,29,31−35^ This increased structural complexity further exemplifies the need to better understand the chemistry that impacts the formation of these UOH/F systems.

While minerals containing uranium and 3d transition metals have been identified, despite their abundance in the Earth’s crust, there is a dearth of UOH minerals containing such species. This has been reasoned as possibly due to uranium-based minerals not being exposed to alteration conditions in the presence of such elements when proceeding along their paragenetic sequence.^15,36^ However, in geological repositories, exposure to such elements and conditions would be much more likely to take place. In addition, transition metals will also be present in both the confinement vessels used for SNF storage and likely in the confinement matrix itself. Therefore, an understanding of the interaction of UOH phases with transition metals is critical.

There have been a small number of reported synthetic UOH compounds containing 3d transition metals (Table 1); however, with only a select few detailed crystal structures available, understanding the role of the transition metal cations in supporting the UOH structure has not been well established. One such 3d transition metal that warrants further study is zinc, with only the one reported study by Chernorukov et al. This sole example of a Zn-containing UOH material stands out given the 290 reported minerals containing Zn, 6 of which have been reported to contain both zinc and uranium.^37^ Interestingly, in each of these 6 minerals, the U/Zn ratio is 1:1 or lower, with no reported structures containing uranium as the majority cation species. Given this would be the case in UOH-based compounds, the ability to study such materials is critical to provide unique insights into the uranium–zinc interactions which may occur in a geological repository setting.

**Table 1:** Synthetic Uranium Oxide Hydrate Materials Containing Transition Metals

UOH–TM2^2+^	chemical formula	space group	crystal structure?
UOF–KCd^32^	Cd_1_K_5_(H_2_O)_6_[(UO_2_)_22_O_23_(OH)_5_]	*C*2*/c*	yes
UOH–KCd^32^	Cd_3_K_2_[(UO_2_)_6_O_9_(OH)_2_]	*Pnnm*	yes
UOH–KNi^28^	K_4_Ni(OH)_3_(H_2_O)_9_[(UO_2_)_12_O_7_(OH)_13_]	*P*3_1_*c*	yes
UOH–KCo^28^	K_4_Co(OH)_3_(H_2_O)_9_[(UO_2_)_12_O_7_(OH)_13_]	*P*3_1_*c*	yes
UOH–Ni^40^	[Ni(H_2_O)_4_]_3_[U(OH,H_2_O)(UO_2_)_8_O_12_(OH)_3_]	*P*1̅	yes
UOH–M (*M* = Mn, Zn, Ni, Co)^41^	[Mn(H_2_O)_4_][(UO_2_)_3_O_3_(OH)_2_]·H_2_O	*P*1̅	yes

An additional consideration is the added presence of K^+^ ions as reported by Zhang et al. in the synthesis of UOH–KNi and UOH–KCo,^28^ along with other UOH compounds containing 2+ metal ions reported to also incorporate Na^+^ or K^+^.^32,38^ From this comes the need to better comprehend the role that monovalent cations play when introduced into these structures alongside 3d transition metals. Broadening this concept beyond Na^+^ and K^+^, cesium (Cs^+^), given its presence in SNF systems as the long-lived ^135^Cs isotope and evidence that it can be incorporated into uranium alteration products, also warrants additional study in this area.^31,39^

In this study, we report the syntheses of two novel UOH/F materials containing Zn^2+^ ions and explore the intricacies of their structures using structural and spectroscopic techniques. **UOHF-Zn** was isolated as a framework-type structure, with Zn^2+^ ions lying within the channels of the uranium backbone in a novel arrangement. In comparison, **UOH-Zn** was found to form a layered UOH-type material with both Zn^2+^ and Cs^+^ cations present as the interlayer cation species, with the Cs^+^ and Zn^2+^ ions acting as pillars, resulting in a pseudoframework type structure. With similar reaction conditions employed in both syntheses, changing the base from KOH for **UOHF-Zn** to CsOH for **UOH-Zn** gave rise to markedly different structures, highlighting the impact that additional monovalent cations present during formation can have on the resultant structure. In addition to exploring these structural features in detail, the microstructures and spectroscopic properties of both compounds were explored by using scanning electron microscopy and powder X-ray diffraction along with Raman and diffuse reflectance spectroscopies.

## 2 Experimental Section

### 2.1 Synthesis of Materials

Uranyl nitrate hexahydrate with natural isotopic ratios of uranium was used in the synthesis of the materials. Compounds with uranium are radioactive and should be handled in a regulated laboratory. All other chemicals of A.R. grade were from Sigma-Aldrich (Merck).

#### 2.1.1 Zn_2_(OH)_2_(H_2_O)_5_[(UO_2_)_10_UO_14_(H_2_O)_3_] (UOHF-Zn)

Zinc nitrate hexahydrate, Zn(NO_3_)_2_·6H_2_O (0.0982 g, 0.33 mmol), and uranyl nitrate hexahydrate (0.165 g, 0.33 mmol) were dissolved in 5 mL of deionized (DI) water, followed by adjustment of the solution pH with KOH until the solution pH was 6.49. The solution was then transferred into a 30 mL Teflon vessel, sealed in a steel autoclave, and heated in an oven at 240 °C for 48 h. Small plate-like crystals of the compound **UOHF-Zn** were obtained after cooling to room temperature at 5 °C/h with the final solution pH of 5.83. Upon washing with DI water and drying in air at ambient temperature, a yield of ∼51 wt % (0.053 g) was isolated. Upon reaction completion, the reaction solution was still pale yellow in color, indicative of either unreacted uranium or water-soluble uranium byproducts also present in the final reaction solution. A decrease of the final solution pH to a pH range of 4 to 5.5 gave rise to a significant portion of amorphous powder and a very fine crystalline secondary phase, which was unable to be characterized further.

#### 2.1.2 Cs_2_Zn(H_2_O)_4_[(UO_2_)_4_O_3_(OH)_4_]_2_·3H_2_O (UOH-Zn)

Zinc nitrate hexahydrate, Zn(NO_3_)_2_·6H_2_O (0.0491 g, 0.165 mmol), and uranyl nitrate hexahydrate (0.0823 g, 0.165 mmol) were dissolved in 10 mL of deionized (DI) water, followed by adjustment of the solution pH with CsOH until the solution pH was 6.57. The solution was then transferred into a 30 mL Teflon vessel, sealed in a steel autoclave, and heated in an oven at 240 °C for 48 h. Small rod-like crystals of the compound **UOH-Zn** were obtained after cooling to room temperature at 5 °C/h with the final solution pH of 5.85. Upon washing with DI water and drying in air at ambient temperature, a yield of ∼63 wt % (0.037 g) was isolated. Upon reaction completion, the reaction solution was still pale yellow in color, indicative of either unreacted uranium or water-soluble uranium byproducts also present in the final reaction solution. Adjustment of the final solution pH below 5.25 and above 6 gave rise to a major amorphous phase and a fine crystalline minor phase, which was unable to be characterized further.

### 2.2 Characterizations

#### 2.2.1 Synchrotron Single Crystal X-Ray Diffraction

Suitable single crystals were harvested as either thin plates (**UOHF-Zn**: ∼29 × 15 × 0.8 μm) or small needles (**UOH-Zn**: ∼20 × 1.2 × 1.0 μm). Data collections for such challenging single crystals are not possible using a lab X-ray diffractometer, and as such, synchrotron X-ray diffraction was utilized.

The single crystal data for compounds **UOHF-Zn** (CCDC-2339546) and **UOH-Zn** (CCDC-2339545) were collected at 100(2) K on the MX2 beamline^42^ at the Australian Synchrotron employing silicon double crystal monochromated synchrotron radiation (λ = 0.71079 Å). Data integration and reduction were undertaken with XDS.^43^ Absorption corrections were applied to the data using SADABS.^44^ The structures were solved by direct methods^45^ and refined with SHELXL-2014^46^ using the Olex2 graphical user interface.^47^ All but hydrogen atoms were located on the electron density maps and refined anisotropically. The structure of **UOH-Zn** was refined using a two-component inversion twin with a Flack of 0.66(3). For the structure refinement of **UOHF-Zn**, one U atom (U5) has an abnormal elongated thermal ellipsoid, and all U atoms want to be lower than unity, likely due to the ineffective absorption corrections. In addition, some oxygen atoms also show small ellipsoids even with NDP (O18) likely due to the ineffective absorption corrections. Therefore, EADP was used to refine all of the oxygen atoms. The one-circle goniometer setup, a low beam attenuation applied to prevent crystal damage, and the poor diffraction at certain angles lead to the low data completeness and ineffective absorption corrections reflected as ripples around heavy U atoms.

#### 2.2.2 Scanning Electron Microscopy (SEM)

The crystal morphologies and elemental compositions were analyzed by using SEM coupled with energy dispersive spectrometry (EDS). Samples were carbon coated and examined in a Zeiss Ultra Plus scanning electron microscope (Carl Zeiss NTS GmbH, Oberkochen, Germany) operating at 15 kV equipped with an Oxford Instruments X-Max 80 mm^2^ SDD X-ray microanalysis system. EDS point analyses were carried out on relatively flat crystal surfaces with a Cu standard for calibration.

#### 2.2.3 Raman Spectroscopy

Raman spectra were collected on a Renishaw inVia spectrometer equipped with a 785 nm excitation Ar laser from 2000 to 100 cm^–1^ with a spectral resolution of ∼1.7 cm^–1^.

#### 2.2.4 Diffuse Reflectance Spectroscopy (DRS)

Absorption spectra in the UV–visible region was recorded on an Agilent Cary 5000 spectrophotometer equipped with a Labsphere Biconical Accessory and referenced to a Labsphere certified standard.

#### 2.2.5 Powder X-Ray Diffraction

Powder X-ray diffraction patterns were collected on a Bruker D8 Focus diffractometer equipped with Cu Kα (λ = 1.5418 Å) radiation in the angle range of 5–90° 2θ with a step size of 0.02° (2θ) and a data collection time of 3 s per step. The collected data were fit to their respective phases via the Le Bail method using TOPAS.^48^

## 3 Results and Discussion

### 3.1 Material Synthesis and Microstructure Investigation

Crystals of **UOHF-Zn** and **UOH-Zn** were successfully isolated after synthesis under hydrothermal conditions with uranyl and zinc nitrates as the metal sources. Prior to heating, adjustment of the pH to 6.49 was performed using KOH for **UOHF-Zn**, with a final solution pH of 5.83 obtained after synthesis. The hydroxide source was changed to CsOH for **UOH-Zn**, wherein the starting solution pH was adjusted to 6.57, resulting in a final solution pH of 5.85. The choice of pH was driven by recent successes in incorporating 2+ secondary cations at higher pH values,^32,38^ with further consideration also that, upon addition of more of the hydroxide, there would also be increased proportions of K^+^ and Cs^+^ in solution. Of note also is that the change in reaction volume between the two syntheses results in slight changes in both reactant concentrations and reaction pressures. Previous studies reveal that a larger reaction volume may impart a level of influence on the formation of a layered-type structure over that of a framework.^34,49^

**Figure 1:**
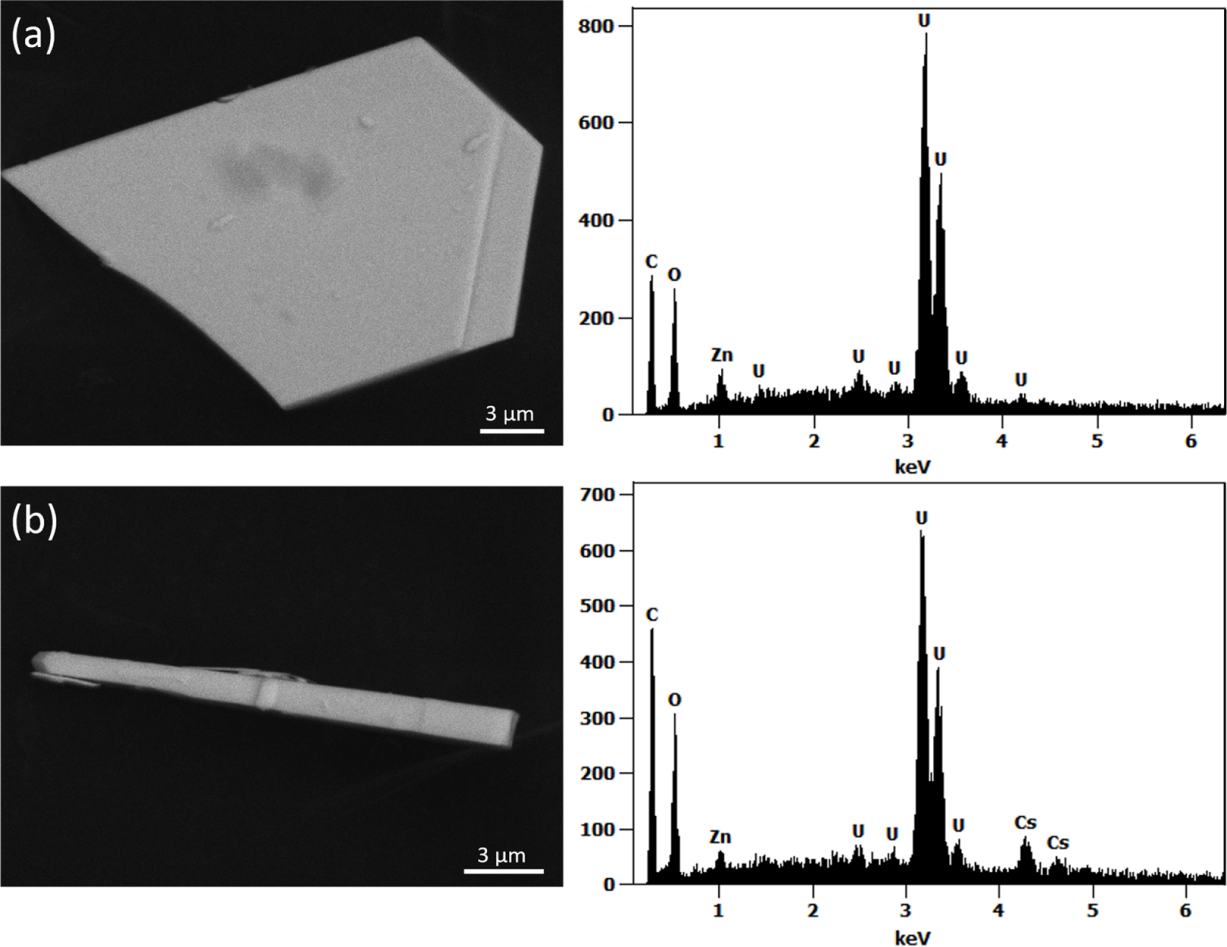
SEM analysis of **UOHF-Zn** (a) and **UOH-Zn** (b): backscattered SEM images of the crystals on the left and their corresponding EDS spectra on the right, confirming the presence of U and Zn (a) and U, Zn, and Cs (b) in the crystals.

SEM-EDS analysis (Figure 1a) revealed a plate-like crystal morphology for **UOHF-Zn**, with EDS analysis confirming the successful incorporation of U and Zn in an atomic ratio of 5.5:1. Notable was the absence of any K in the structure. Upon examination of **UOH-Zn** with SEM (Figure 1b), a distinctly different crystal morphology was identified, with needle-like crystals instead observed. EDS analysis revealed that both Zn and Cs had been successfully incorporated into the structure, with a U/Zn/Cs ratio of 8:1:2. Interestingly, the atomic ratios closely matched that of a published UOH-Mg compound, wherein crystals containing a U/Mg/Na ratio of 8:1:2 were obtained in similar needle-like crystals.^38^

In both syntheses, a similar minor phase consisting of hexagonal plate crystals was also identified (Figure S1), with EDS analysis confirming that the material only contained U and O, with no secondary cations present. Thus, this minor phase was not explored further. Separation of this minor phase was performed, following which powder X-ray diffraction was performed to establish that the bulk material was phase pure and matched the calculated pattern obtained from single-crystal data. A Le Bail fitting was performed and revealed that **UOHF-Zn** (Figure S2, top) showed a close match between the bulk phase and the calculated pattern, confirming a phase pure compound was obtained for further analysis. Analysis of **UOH-Zn** (Figure S2, bottom) revealed that a slight impurity was present in the bulk material, best evidenced by additional peaks between 25° and 35° 2θ. Given the presence of the minor phase under SEM, this was not unexpected, and it was confirmed that the major phase present closely matched that determined from the single-crystal data and was suitable for further analysis.

### 3.2 Crystal Structures and Discussion

Crystals of suitable quality were isolated for both **UOHF-Zn** and **UOH-Zn**, and single-crystal X-ray diffraction data were obtained by using synchrotron radiation. The crystal data and structure refinement details for **UOHF-Zn** and **UOH-Zn** are summarized in Table 2, with selected bond lengths (Å) and angles (°) provided in Tables 3 and 4, respectively.

**Table 2:** Crystal Data and Structure Refinement Details for Compounds **UOHF-Zn** and **UOH-Zn**

compound	UOHF-Zn	UOH-Zn
CCDC	2339546	2339545
empirical formula	ZnO_22_U_5.5_	Cs_2_ZnO_37_U_8_
formula weight	1726.54	2827.43
crystal system	triclinic	orthorhombic
space group	*P*1̅	*Cmc*2_1_
*a* (Å)	8.2010(16)	14.979(3)
*b* (Å)	10.951(2)	13.885(3)
*c* (Å)	11.510(2)	16.577(3)
α (deg)	111.40(3)	90
β (deg)	102.35(3)	90
γ (deg)	104.24(3)	90
volume (Å^3^)	878.5(4)	3447.9(12)
*Z*/μ (mm^–1^)	2/51.958	4/40.306
min/max θ (deg)	2.018/24.995	2.347/25.000
*d*_calcd_ (g cm^–3^)	6.527	5.447
GOF	1.138	1.049
final *R*_1_^a^ [*I* > 2σ(*I*)]	0.0527	0.0513
final *wR*_2_^b^ [*I* > 2σ(*I*)]	0.1559	0.1135
Flack	-	0.66(3)

a *R*_1_ = Σ∥*F*_o_| – |*F*_c_∥/|*F*_o_|.

b *wR*_2_ = {Σ[*w*(*F*_0_^2^ – *F*_c_^2^)^2^]/Σ[*w*(*F*_0_^2^)^2^]^1/2^.

**Table 3:** Selected Bond Lengths (Å) and Angles (°) for **UOHF-Zn**

UOHF-Zn							
U1–O1	1.794(18)	U2–O3	1.930(18)	U3–O5^a^	2.194(18)	U4–O8	2.263(18)
U1–O2	1.768(18)	U2–O4	1.827(19)	U3–O6	1.849(18)	U4–O8^b^	2.249(19)
U1–O3	2.396(18)	U2–O5^c^	2.279(19)	U3–O7	1.948(19)	U4–O9	2.271(19)
U1–O7^d^	2.344(18)	U2–O5	2.263(18)	U3–O8	2.214(19)	U4–O10	1.838(18)
U1–O11^d^	2.386(18)	U2–O13^a^	2.267(18)	U3–O9	2.174(18)	U4–O11	1.998(18)
U1–O11^a^	2.495(18)	U2–O14^e^	2.421(19)	U3–O18^f^	2.182(19)	U4–O12	2.432(18)
U1–O13^a^	2.354(18)	U2–O18^f^	2.312(18)	O6=U3=O7	174.3(8)	U4–O13	2.161(18)
O2=U1=O1	179.2(9)	O4=U2=O3	176.4(8)			O10=U4=O11	173.9(8)
U5–O12^g^	1.978(18)	U6–O9^h^	2.268(18)	Zn1–O1^a^	2.210(19)	Zn2–O4	2.08(2)
U5–O12	1.978(18)	U6–O12^h^	2.467(19)	Zn1–O6	2.022(19)	Zn2–O16^i^	2.00(2)
U5–O14^g^	1.966(19)	U6–O14^h^	2.468(18)	Zn1–O17	2.010(19)	Zn2–O21	1.89(2)
U5–O14	1.966(19)	U6–O16	1.848(18)	Zn1–O20	1.983(19)	Zn2–O22	2.02(2)
U5–O15^g^	2.298(18)	U6–O17	1.861(18)	Zn1–O21	1.974(19)	Zn2–O23	2.01(4)
U5–O15	2.298(18)	U6–O18^b^	2.282(19)	Zn1–O21^f^	2.33(2)		
O12=U5=O12	180.0(7)	U6–O19	2.1685(10)				
		O16=U6=O17	176.1(8)				

a *X*, *Y*, −1 + *Z.*

b 1 – *X*, 1 – *Y*, 1 – *Z*.

c 1 – *X*, 2 – *Y*, 1 – *Z*.

d –*X*, 2 – *Y*, 1 – *Z.*

e –*X*, 1 – *Y*, 1 – *Z*.

f 1 – *X*, 1 – *Y*, 1 – *Z*.

g –*X*, 1 – *Y*, 2 – *Z*.

h 1 + *X*, *Y*, *Z*.

i 2 – *X*, 1 – *Y*, 1 – *Z*.

**Table 4:** Selected bond lengths (Å) and angles (deg) for **UOH-Zn**

UOH-Zn							
U1–O1	1.79(2)	U2–O3^a^	2.45(2)	U3–O5	2.43(2)	U4–O3^b^	2.52(2)
U1–O2	1.77(2)	U2–O5	2.52(2)	U3–O6^b^	2.26(2)	U4–O4	2.27(2)
U1–O3	2.41(2)	U2–O6	2.27(3)	U3–O9^b^	2.51(3)	U4–O9^c^	2.40(2)
U1–O4	2.35(2)	U2–O7	1.82(3)	U3–O10	2.27(2)	U4–O10^d^	2.27(2)
U1–O5	2.44(2)	U2–O8	1.84(3)	U3–O11	1.80(3)	U4–O13	2.44(2)
U1–O6	2.22(2)	U2–O9	2.36(2)	U3–O12	1.81(3)	U4–O14	1.77(3)
U1–O13^e^	2.59(2)	U2–O10	2.23(2)	U3–O13	2.47(2)	U4–O15	1.79(3)
O2=U1=O1	178.7(11)	O7=U2=O8	177.8(11)	O11=U3=O12	176.3(11)	O14=U4=O15	177.5(11)
Cs1–O2^f^	3.12(2)	Cs1–O11^e^	3.15(3)	Cs2–O8^d^	3.19(2)	Zn1–O1	2.19(2)
Cs1–O2	3.12(2)	Cs1–O11^g^	3.15(3)	Cs2–O8^h^	3.19(2)	Zn1–O7	2.05(3)
Cs1–O6	3.43(3)	Cs1–O16^g^	3.31(3)	Cs2–O10^d^	3.22(3)	Zn1–O11	2.23(2)
Cs1–O6^f^	3.43(3)	Cs1–O17^g^	3.13(3)	Cs2–O10^h^	3.22(3)	Zn1–O16	2.19(3)
Cs1–O8^f^	3.23(2)	Cs1–O19	3.74(4)	Cs2–O12^h^	3.33(2)	Zn1–O17	2.11(2)
Cs1–O8	3.23(2)			Cs2–O12^d^	3.33(2)	Zn1–O18	2.06(2)
				Cs2–O14^i^	3.13(2)		
				Cs2–O14	3.13(2)		

a *X*, −*Y* + 1, *Z* – 1/2.

b –*X* + 3/2, *Y* – 1/2, *Z*.

c *X*, −*Y* + 1, *Z* + 1/2.

d –*X* + 3/2, −*Y* + 1/2, *Z* + 1/2.

e –*X* + 3/2, *Y* + 1/2, *Z*.

f –*X* + 3/2, −*Y* + 1/2, *Z* + 1/2.

g *X* + 1/2, *Y* + 1/2, *Z*.

h *X* – 1/2, −*Y* + 1/2, *Z* + 1/2.

i –*X* + 1, *Y*, *Z*.

**UOHF-Zn** was identified as crystallizing in the triclinic *P*1̅ space group with six distinct uranium centers and two zinc centers, each in partial occupancies, composing the asymmetric unit. Extending the structure gives rise to a framework-type structure, with the uranium centers composing the framework backbone and the Zn centers lying in the channels. The uranium sites within the framework take on two distinct geometries, U3 and U5 present in octahedral geometries and the remaining U centers existing as pentagonal bipyramids. The two zinc centers are also in different geometries, with Zn1 having a 6-fold, distorted octahedral geometry and Zn2 taking on a 5-coordinate trigonal bipyramidal orientation.

**Figure 2:**
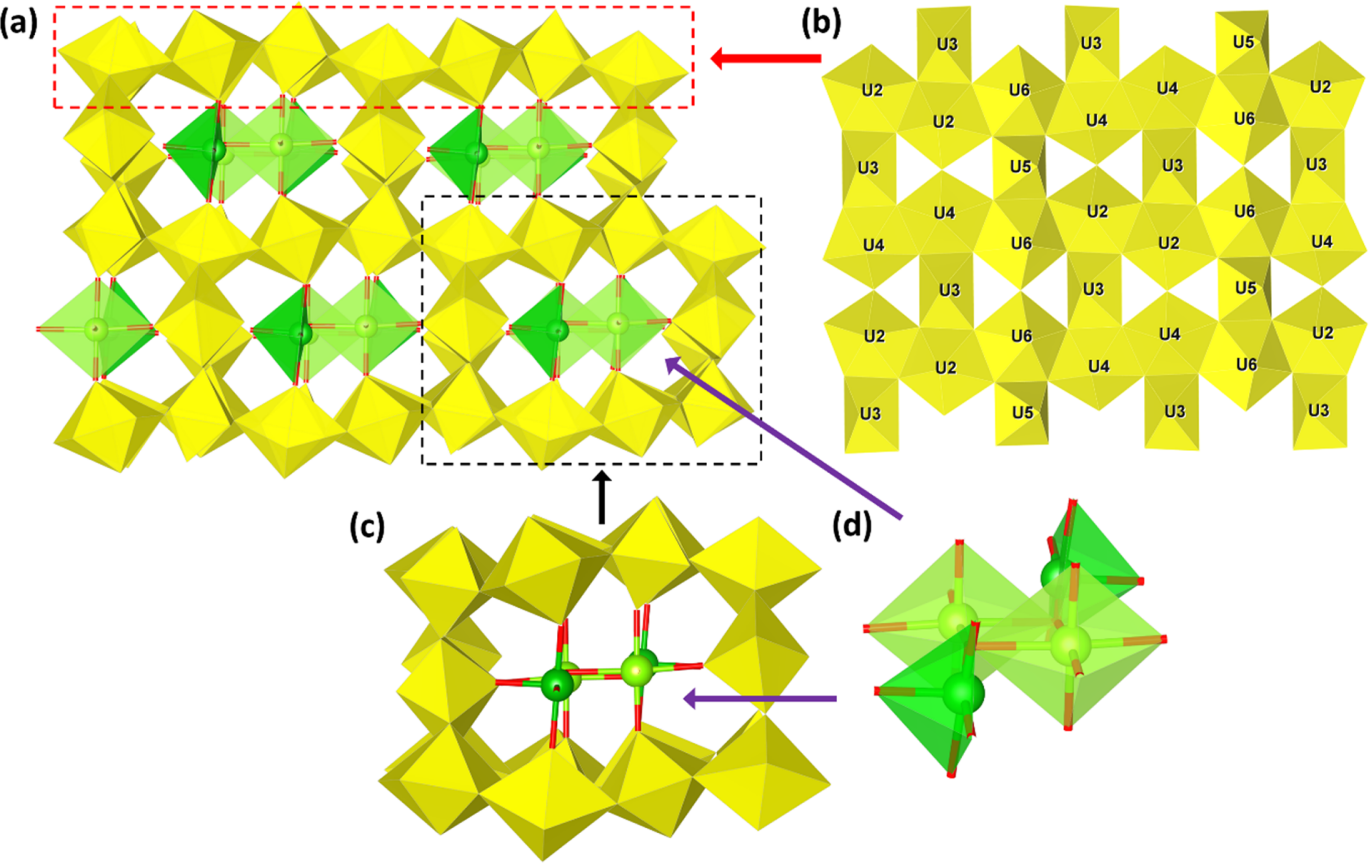
Crystal structure of **UOHF-Zn**: a polyhedral crystal structure along the *c*-axis (a), a polyhedral view of the uranyl oxide hydroxide layer with a β-U_3_O_8_ topology (b), and the 5- and 6-fold coordinated Zn^2+^ centers composing the Zn binding unit and lying within the channels of the framework (c) and (d); U in yellow, 6-fold Zn in dark green, and 5-fold Zn in pale green.

The layout of the uranyl polyhedra sheets is structurally similar to that of β-U_3_O_8_ and those seen in previous UOH/F systems,^33−35^ with chains of U2, U4, and U6 pentagonal bipyramids connected via O–O edge sharing and U3 and U5 octahedra linking the chains together (Figure 2b). The layers are pillared through pairs of U1–U1 units, giving rise to the channels that extend throughout the framework structure. The two octahedral U centers show quite interesting coordination environments, differing significantly from each other. U3 resembles that of a uranyl unit, with four equatorial U–O bonds between 2.174(18) and 2.214(19) Å and two shorter axial bonds of 1.849(18) – 1.948(19) Å in length. The slightly elongated U3–O7 bond arises from the additional coordination of O7 to U1, with this type of cation–cation interaction (CCI) reported to result in a slight elongation of the uranyl bond.^50^

In comparison, U5 lacks the appearance of a uranyl bond. U5 lies coplanar with O12 and O14, with observed bond lengths of 1.978(18) and 1.966(19) Å, respectively, and O12–U5–O12 and O14–U5–O14 bond angles of 180°. The O12–U5–O14 angles are 82.1(8)° and 97.9(8)°, with this uranium center very closely matching a structure reported by Burns and coworkers.^50^ U5 donates double CCIs through both the O12 and the O14 oxygens to neighboring pentagonal bipyramidal uranium centers, with bond lengths of 1.978(18) and 1.966(19) Å matching those expected for such interactions. As such, the U5 site can be defined as having a tetraoxido core. Such uranium centers have also been found in other synthetic systems which have the same β-U_3_O_8_ layer topology; however, in such cases, further discussion of the coordination environment was not mentioned.^32,38,40^ Excluding U5, the remaining uranium centers have bond length and angle values that are consistent with previously reported UOHF materials, including the slight bending of the typically linear uranyl bonds.^29,32,33,35^

Examining the bond valence sum (BVS) calculations (Table S1), using values obtained from the literature,^14^ confirmed that all U centers are present as U^6+^ [U1 (5.96), U2 (5.87), U3 (5.75), U4 (5.91), U5 (5.90), and U6 (5.91)], with Zn^2+^ [Zn1 (2.19) and Zn2 (2.20)] also confirmed for the Zn centers. The majority of the oxygens are present as O (O1–O14, O16–O18) with one OH (O21) and three full occupancy (O15, O20, and O22) and two half occupancy (O19 and O23) H_2_O species, allowing for the formulation of **UOHF-Zn** to be determined as Zn_2_(OH)_2_(H_2_O)_5_[(UO_2_)_10_UO_14_(H_2_O)_3_] (*Z* = 2).

While the uranyl framework is comparable to other UOHF structures, of particular interest, however, is the nature of the Zn^2+^ cations that lie in the channels. **UOHF-Zn** is the first reported UOHF structure incorporating transition metal ions alone, and only the third with solely 2+ cations,^27,29^ and thus their coordination environment is intriguing to explore. Also of note is that the reported space group, that of *P*1̅, has only been reported once previously for a UOHF compound.^33^ Upon examination of the zinc centers, immediately evident is that the Zn^2+^ cations form discrete units that are isolated from one another, each composed of two Zn1 and two Zn2 centers (Figure 2d). Two Zn1 moieties make up the center of this unit, connected through the O–O edge sharing (O21), with the two Zn2 centers projecting into the channels and linked via corner sharing through the O21. These Zn units are comparable to the binding motif seen for UOHF-Er/Y,^33^ wherein the interchannel cations exist isolated from their neighboring cations, as opposed to forming chains that extend along the channels of the framework as seen in other UOHF structures.^34,35^ Given that the *P*1̅ space group found for **UOHF-Zn** is the same as that reported in the UOHF-Er/Y study, this similarity is logical. In contrast to that study, however, is the existence of a Zn binding unit in **UOHF-Zn** in place of the singular Er/Y^3+^ cation. The most likely explanation for this notable difference is the ionic radii of Zn^2+^ (0.68–0.74 Å), which is markedly smaller than that of Er/Y^3+^ (1.004 Å).^51^ Thus, it makes sense that to bind within channels with comparable dimensions, a different binding motif must be adopted. Related to this consideration is coordination between the Zn unit and the uranyl framework. While each of the Zn centers is coordinated to the uranyl polyhedra layers through the axial U–O bonds, Zn1 also coordinates to the U1 pentagonal bipyramids making up the “walls” of the channels through the axial oxygens (O1), anchoring the Zn unit to all four sides of the framework (Figure 2c), a unique feature heretofore unseen in any UOHF structure. A second aspect that may also drive the observed changes in structure from those previously reported, likely in tandem with the change in ionic radius, is the 2+ charge on the Zn centers. However, these structural features are also unique compared to those seen for Cd^2+^, Pb^2+^, and Sr^2+^,^27,29,32^ which are each significantly larger in ionic radius compared to Zn^2+^.

In comparison to the previously reported UOHF-Cd structure,^32^ K^+^ is notably absent in **UOHF-Zņ** despite comparable synthetic conditions being used in both syntheses. The smaller ionic radius of Zn^2+^ is again likely to have played a role in this result, providing additional freedom for the secondary cation to be incorporated and satisfying the charge balance of the structure without the need for K^+^ to also being present. Previously hypothesized,^19,32^ and clearly highlighted by **UOHF-Zn**, is the fact that the ionic radius of the secondary cation is a critical aspect to consider when examining these materials.

**Figure 3:**
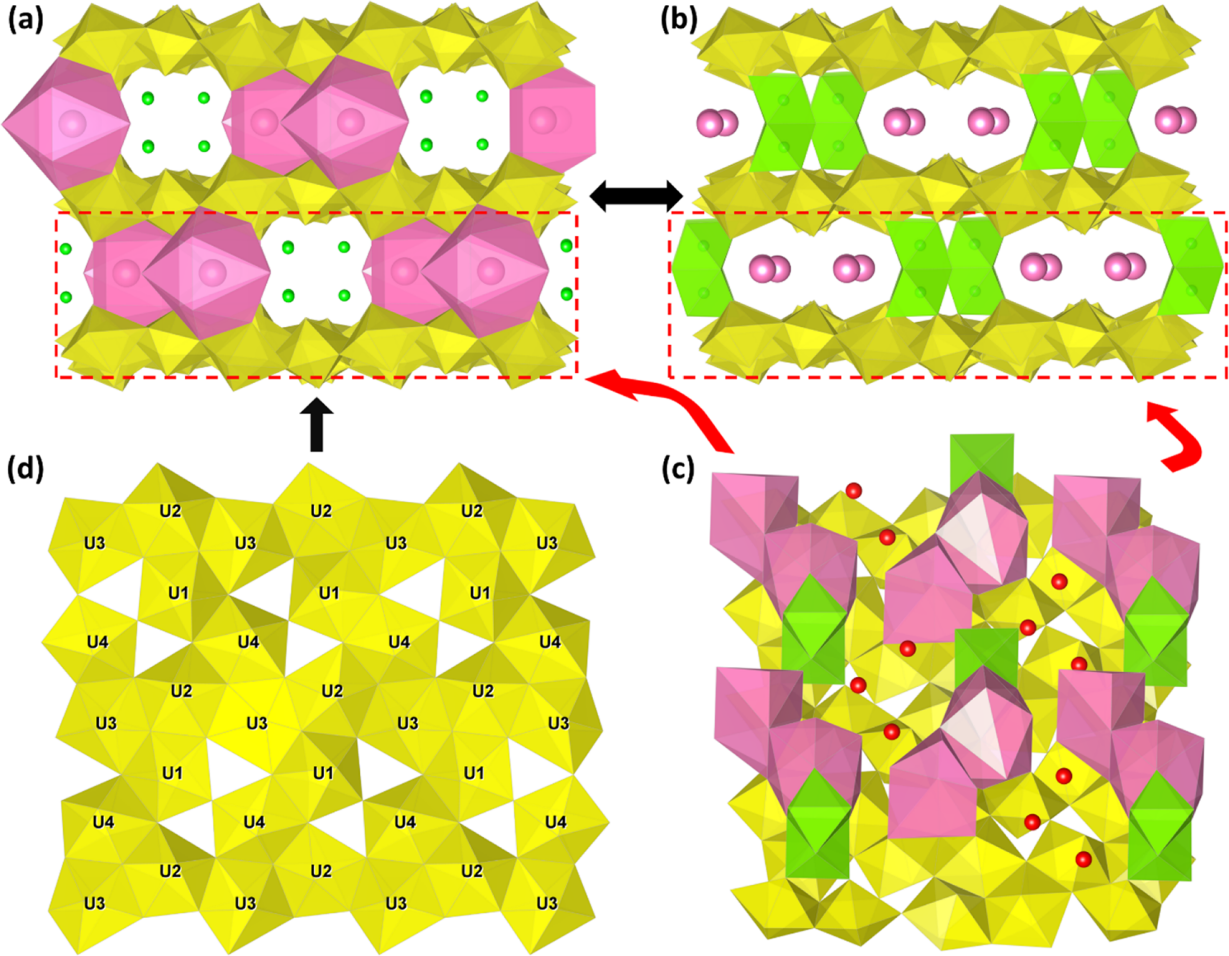
Crystal structure of **UOH-Zn**: a polyhedral crystal structure along the *c*-axis with Zn^2+^ shown as balls (a), a polyhedral crystal structure along the *c*-axis with Cs^+^ shown as balls (b), a polyhedral view illustrating the Cs–Zn “pillars” and H_2_O molecules along the *b*-axis (c), and a polyhedral view of the uranyl oxide hydroxide layer with a schoepite-like layer topology (d); U in yellow, Cs in pink, and Zn in green.

Examining the crystal structure of **UOH-Zn** revealed that it crystallized in the orthorhombic space group *Cmc*2_1_. Confirming the results found with SEM-EDS (Figure 1b), the asymmetric unit was found to contain two half occupancy Cs centers along with one-half occupancy Zn site and four full occupancy U centers, confirming the elemental ratio of 2:1:8. All four U sites take on the same pentagonal bipyramidal geometry, with the Zn existing in a distorted octahedral geometry. The two Cs sites differ significantly, with an 11-coordinate all faced capped trigonal prism (Cs1) and an 8-fold distorted bicapped trigonal prismatic (Cs2) both linked through O–O edge sharing. In contrast to **UOHF-Zn**, the uranium oxide hydroxide layer instead more closely resembled that of schoepite (Figure 3d).^52^ Chains of −U3–U2– centers are linked through the U1 and U4 centers via the O–O edge and corner sharing, with the axial oxygens projecting above and below the layer and participating in the uranyl–cation interactions.

The coordination sphere about each of the U sites shows the expected uranyl bonds in the axial positions (1.77(2)–1.84(3) Å), with slightly distorted U=U=O uranyl bond angles of 176.3(11)°–178.7(11)°. The equatorial U–O bonds fall in the range of 2.22(2)–2.59(2) Å, consistent with the uranyl centers in previously reported structures.^28,30,39^ Each of these uranyl polyhedra layers are separated by a distance of ∼6.8 Å, within which lie the secondary cations. The BVS calculations (Table S2) confirmed that all U centers are present in the U^6+^ state [U1 (5.98), U2 (5.85), U3 (5.88), and U4 (6.07)], with the Cs centers existing as Cs^+^ [Cs1 (1.19) and Cs2 (0.94)] and the Zn center as Zn^2+^ (1.86). Four OH (O3, O4, O5, and O13), four half occupancy H_2_O (O16, O17, O19 and O20), and three half occupancy unbound H_2_O molecules (O20–O22) are all present, and thus **UOH-Zn** was formulated as Cs_2_Zn(H_2_O)_4_[(UO_2_)_4_O_3_(OH)_4_]_2_·3H_2_O (*Z* = 4).

Of immediate interest when examining the interlayer cations is the dual-cation system, as monovalent cations have been identified as an additional means of providing a charge balance to the uranium oxide hydroxide layer. As such, introducing alkali metals that will be present in the surroundings of SNF in a deep geological setting, such as Na^+^, K^+^ and Cs^+^, may provide further insight into the stability and flexibility of the alteration products of uranyl species. Notably, the presence of both Cs^+^ and Zn^2+^ in the interlayer of **UOH-Zn** is the first reported UOH material with Cs^+^ present in a dual-cation system. The binding of these cations is comparable to that of Zn^2+^ in **UOHF-Zn** in that the Cs^+^ and Zn^2+^ form isolated binding units, in this case consisting of one center each of Cs1 and Cs2, along with two Zn2 centers (Figure 3b). The two Zn centers coordinate each other and Cs1 through O–O edge sharing, accounting for the increased coordination number for Cs1 compared to Cs2. These binding units are then bound to each uranyl layer again through uranyl–cation interactions. The result is isolated Cs–Zn “pillars”, with void space lying between each and giving rise to a pseudoframework structure. Within these voids can be found one bound (O19) and three unbound (O20–O22) water molecules (Figure 3b). This pseudoframework is comparable to structures published by Zhang et al.^49^ and Rivenet et al.;^40^ however, a notable difference is that in each of those studies, the “pillar” consisted of a singular ion, where in **UOH-Zn**, the pillar is made up of the Cs–Zn binding unit.

An interesting comparison can also be made to another recent study by Zhang et al.^38^ In that study, a dual-cation interlayer of Na^+^ and Mg^2+^ was reported, termed **UOH-Mg2n**, and was found to have an identical atomic ratio of 2:1:8 (Na/Mg/U), with the same molecular formula as that reported for **UOH-Zn**. Notably different, however, was that **UOH-Mg2n** crystallized in the *P*1̅ space group and was found to have a novel uranium oxide hydroxide layer compared to the schoepite-like topology reported for **UOH-Zn**. In examining the two materials, what is apparent is both the near identical ionic radii of Mg^2+^ and Zn^2+^ (0.72–0.74 Å) and the increased ionic radius of Cs^+^ compared to Na^+^ (1.67–1.88 Å vs 1.02–1.39 Å).^51^ While additional factors must also be considered, such as the influence of the solution pH and the binding nature of the other cation species (being Mg^2+^ and Zn^2+^), what these two studies further emphasize is the intricate balance of all components within the synthesis of these materials, and as such, the multifaceted nature of the alteration of uranium oxide materials when placed within a geological repository.

### 3.3 Electronic Structures

**Figure 4:**
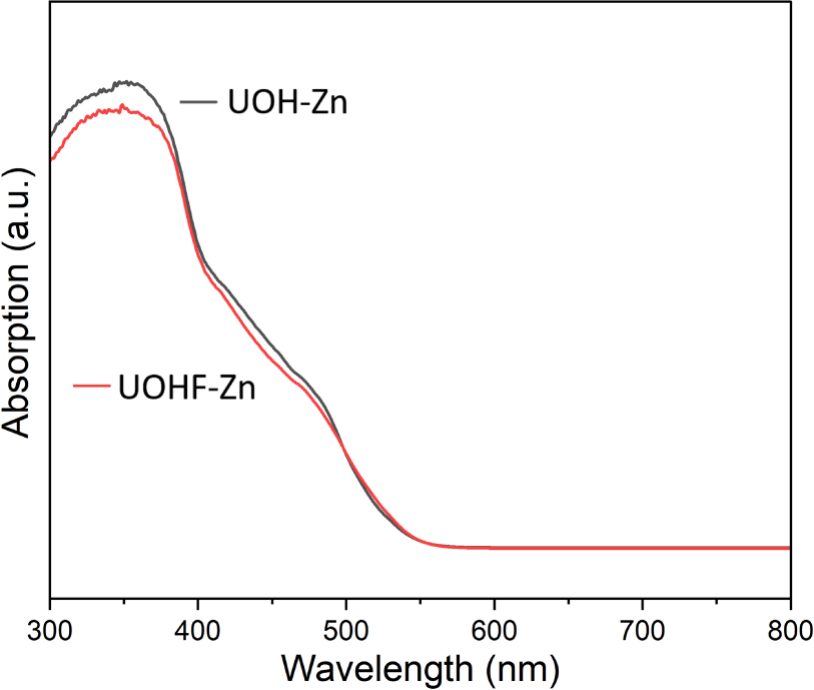
DR spectra of **UOHF-Zn** (red) and **UOH-Zn** (gray) in the UV–vis region.

The calculated BVS values for both materials (Tables S1 and S2) indicate that only U^6+^ is present, unlike previously reported UOH/F structures.^27,33,53^ The dominating feature of both DR spectra (Figure 4) is the broad absorption peaks in the UV region (300–500 nm), with peaks at ∼350 nm and a possible obscured peak at ∼460 nm, as indicated by a shoulder, arising from charge-transfer bands from the U^6+^ centers.

### 3.4 Vibrational Modes

**Figure 5:**
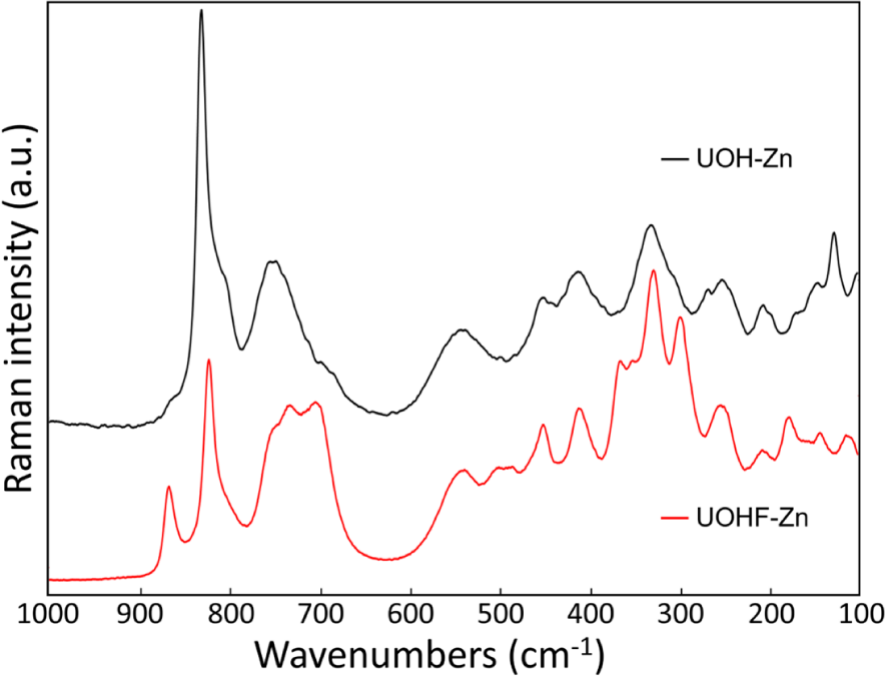
Raman spectra of **UOHF-Zn** (red) and **UOH-Zn** (black).

Raman spectroscopy was employed to explore the vibrational modes of **UOHF-Zn** and **UOH-Zn**. The spectra for both materials are comparable (Figure 5), expected given the similar nature of their uranium oxide hydroxide layers. Peaks in the region of 900–650 cm^–1^ can be assigned to ν_1_(UO_2_)^2+^ vibrations corresponding to the uranyl U=O bonds. Given the variety of unique U centers and the U=O bond lengths spanning a broad range (1.768–1.98 Å), multiple ν_1_(UO_2_)^2+^ vibrations are evident in both spectra. Additional peaks at 865 and 700 cm^–1^ are evident in the **UOHF-Zn** spectrum, expected given the two additional 6-fold U(VI) centers. These values are broadly consistent with the values reported for U=O bonds in the literature.^54,55^

The broad, medium intensity peaks between 580 and 320 cm^–1^ arise from γ[U_3_(OH)_3_] bending vibrations, ν(U_3_O) bridge elongations, and ν(U–O_ligand_) vibrations, with the remaining peaks assigned to ν_2_(UO_2_)^2+^ bending vibrations (295–200 cm^–1^) and lattice vibrations (180–100 cm^–1^).

### 3.5 Implications and Perspectives

The capacity to synthesize two novel UOH/F materials under similar reaction conditions, with only variations on the concentrations and identity of the cation species, clearly demonstrates the value of synthetic UOH compounds in identifying and studying the possible alteration products of uranium oxides in a geological setting. The abundance of transition metals in the Earth’s crust guarantees that these species will be present in the surroundings of any deep geological repository, yet due to paragenesis, no such UOH minerals are available for study, driving the need to make such phases synthetically. However, the lack of reported UOH materials containing transition metals^28,32,40,41^ makes understanding the influence of 3d/4d transition metals on the alteration pathway of uranium oxides challenging. In successfully incorporating a small 3d transition metal into a UOHF structure, the scope of what elements may adopt this phase along the alteration pathway is significantly broadened, highlighting the complexity of these systems. The novel binding motif adopted by the Zn^2+^ cations in the channels of the framework, again governed by the smaller ionic radius compared to cations previously reported, provides critical insight into the possible options available to meet both the essential size and charge balance required to be incorporated into these materials.

With only a few examples of synthetic UOH structures containing multiple interlayer cation species, despite the much larger number of UOH minerals containing such systems, there is a clear gap in knowledge that must be filled. Through the successful incorporation of both Zn^2+^ and Cs^+^ cations into a layered UOH structure, the first time Cs^+^ has been incorporated alongside a second cation species in such a material, this knowledge gap is clearly exemplified. The abundance of Cs in SNF, along with the abundance of Na and K in the surroundings of any geological repositories, makes understanding these systems better a clear priority. Also highlighted within this work was the exclusion of K^+^ from the UOHF structure despite comparable reaction conditions. The additional factors of reactant concentration and reaction pressure were also considered via the adjustment of the total reaction volume. However, in comparing both **UOHF-Zn** and **UOH-Zn** to similar structures in the literature,^32,38^ which, while having very similar reaction conditions, have drastically different crystal structures, we can elucidate that the predominant driving force behind such changes is very likely to be that of the ionic radii of the secondary cation species.

Invaluable also is the means by which these materials are examined crystallographically. In isolating crystals of synthetic UOH phases containing either Zn^2+^ or Zn^2+^ and Cs^+^ cations, it was possible to explore the subtle differences in their crystal structures compared to others previously reported. Highlighting the value of this is the comparison between the herein reported Zn^2+^/Cs^+^ layered UOH structure to that of a published UOH with Mg^2+^/Na^+^ ions, which despite having identical atomic ratios and chemical formulas, had distinct differences in their respective crystal structures that may have otherwise remained unobserved. Further work toward growing and isolating crystals of high enough quality to obtain single crystal X-ray data is therefore critical to gaining a deeper understanding into these materials.

## 4 Conclusions

Two novel UOH/F materials containing Zn^2+^ or Zn^2+^ and Cs^+^ have been successfully synthesized hydrothermally with solution pH controlled through the addition of dilute solutions of KOH and CsOH, respectively. The first of the two structures, **UOHF-Zn**, is a framework-type structure and crystallized in the *P*1̅ space group, with Zn^2+^ cations lying within the channels. This is the first example of small 3d transition metals being successfully incorporated into a UOHF material with the ionic radius of the Zn^2+^ ion significantly smaller than any previously reported inside a framework-type compound. The second structure, **UOH-Zn**, was solved in the *Cmc*2_1_ space group and consisted of schoepite-like UOH layers with Cs^+^ and Zn^2+^ cations making up the interlayer cations with a U/Zn/Cs ratio of 8:1:2. The schoepite-like UOH layer differed from that observed for **UOHF-Zn**, which instead had a uranium oxide hydroxide layer resembling that of β-U_3_O_8_. Interestingly, the Zn^2+^ and Cs^+^ cations formed novel “pillars”, which separated the uranyl layers and resulted in a pseudoframework structure, with both bound and unbound water lying in the framework voids. Unfortunately, removal of these waters has previously been found to result in a loss of crystallinity, indicative of a complete collapse of the structure.^56^ As such, these voids were unable to be accessed for additional study, as any removal of either the cocoordinated or solvent water molecules during the activation and degassing processes for gas absorption would result in structural collapse.

Also of note was the dual-cation system and the first time that Cs^+^ has been successfully incorporated alongside a second cation in such materials.

Significantly, the differences between the two structures despite the synthetic conditions closely resembling one another, except for the identity of the base and the reaction volume, highlight the sensitivity of these materials to their immediate environment during formation. Additionally, the ionic radii of the cations being incorporated within these structures were highlighted as playing a critical role in driving the formation and alteration of these UOH/F systems. From this study, further research is therefore warranted to explore the capability of 3d transition metals to be incorporated into these systems, with additional studies exploring the effect of dual-cation systems on the final product also needed to better understand the chemistry driving the formation of these materials.

## Supporting Information Available

SEM-EDS data; PXRD data; BVS calculations (PDF)
CCDC-2339546 (**UOHF-Zn**) (CIF)
CCDC-2339545 (**UOH-Zn**) (CIF)

## Supplementary Material

ao4c06188_si_001.pdf

ao4c06188_si_002.cif

ao4c06188_si_003.cif

## Abbreviations

BVS: bond valence sum
DR: diffuse reflectance
EDS: energy dispersive spectrometry
SEM: scanning electron microscopy
SNF: spent nuclear fuel
UOH: uranium oxide hydrate
UOHF: uranium oxide hydrate framework
