# Supplementary material for: Incorporating Transition Metal Ions into Uranium Oxide Hydrates: The Role of Zn(II) and the Effect of the Addition of Cs(I) Ions
Source: ACS Omega. 2024 Aug 23;9(36):38284–94. doi: 10.1021/acsomega.4c06188 (PMC11391564; doi:10.1021/acsomega.4c06188)
Supplement: Supplementary file 1 — ao4c06188_si_001.pdf [file ao4c06188_si_001.pdf]

Supplementary Information

Incorporating transition metal ions into uranium oxide hydrates: the role of Zn(II) and the effect of the addition of Cs(I) ions

Timothy A. Ablott*, Kimbal T. Lu, and Yingjie Zhang

Australian Nuclear Science and Technology Organisation, Locked Bag 2001, Kirrawee DC, NSW 2232, Australia.

* Corresponding author: *ablottt@ansto.gov.au* (T. Ablott)

Key words: uranium oxide hydrate, transition metal, zinc, cesium, structure, spectroscopy

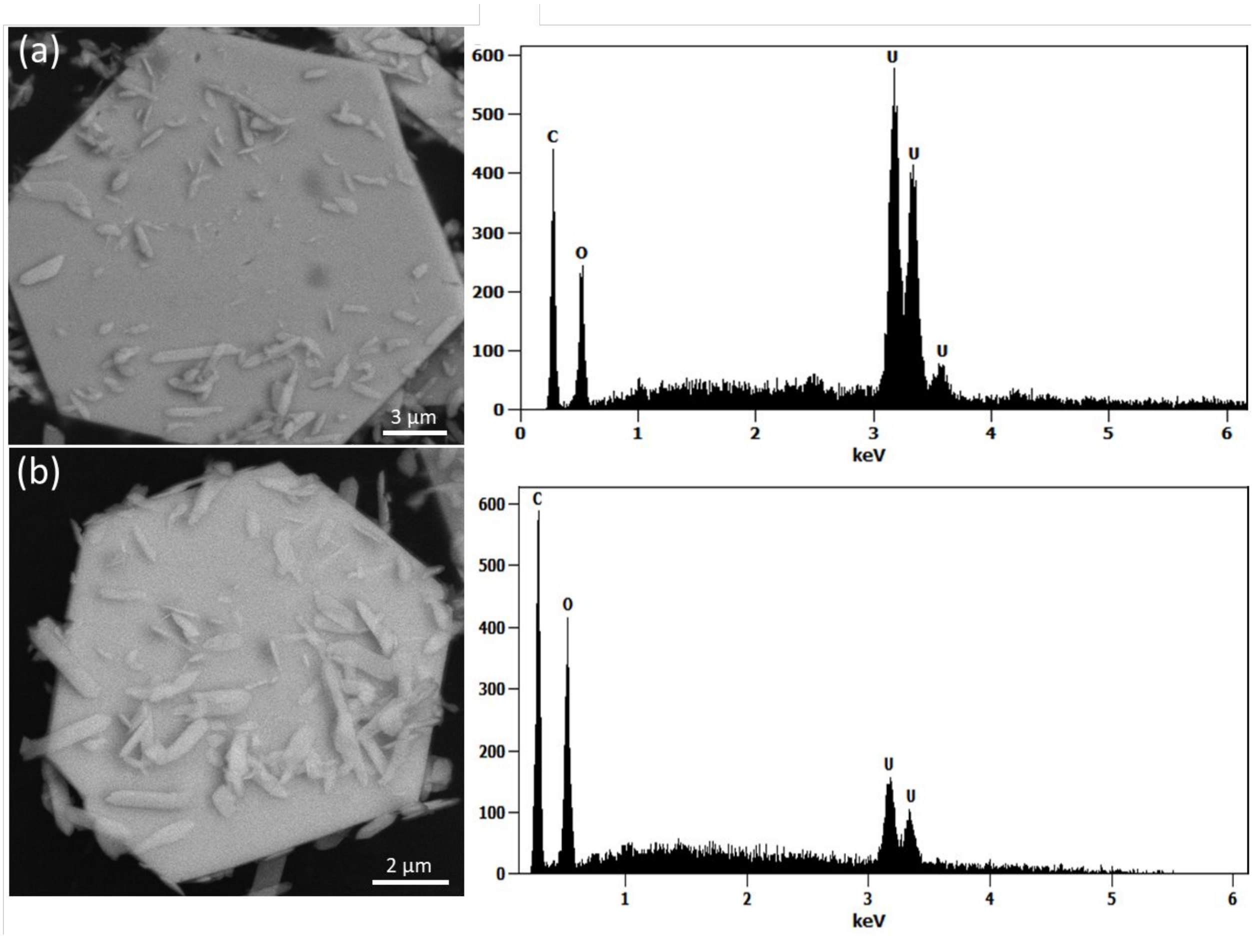

Figure S1. SEM-EDS of the minor phase isolated from the synthesis of both **UOHF-Zn** and **UOH-Zn**: an SEM image (a) and the corresponding EDS spectrum (b) of the hexagonal plate-like crystals confirming the presence of only U and O in the crystal.

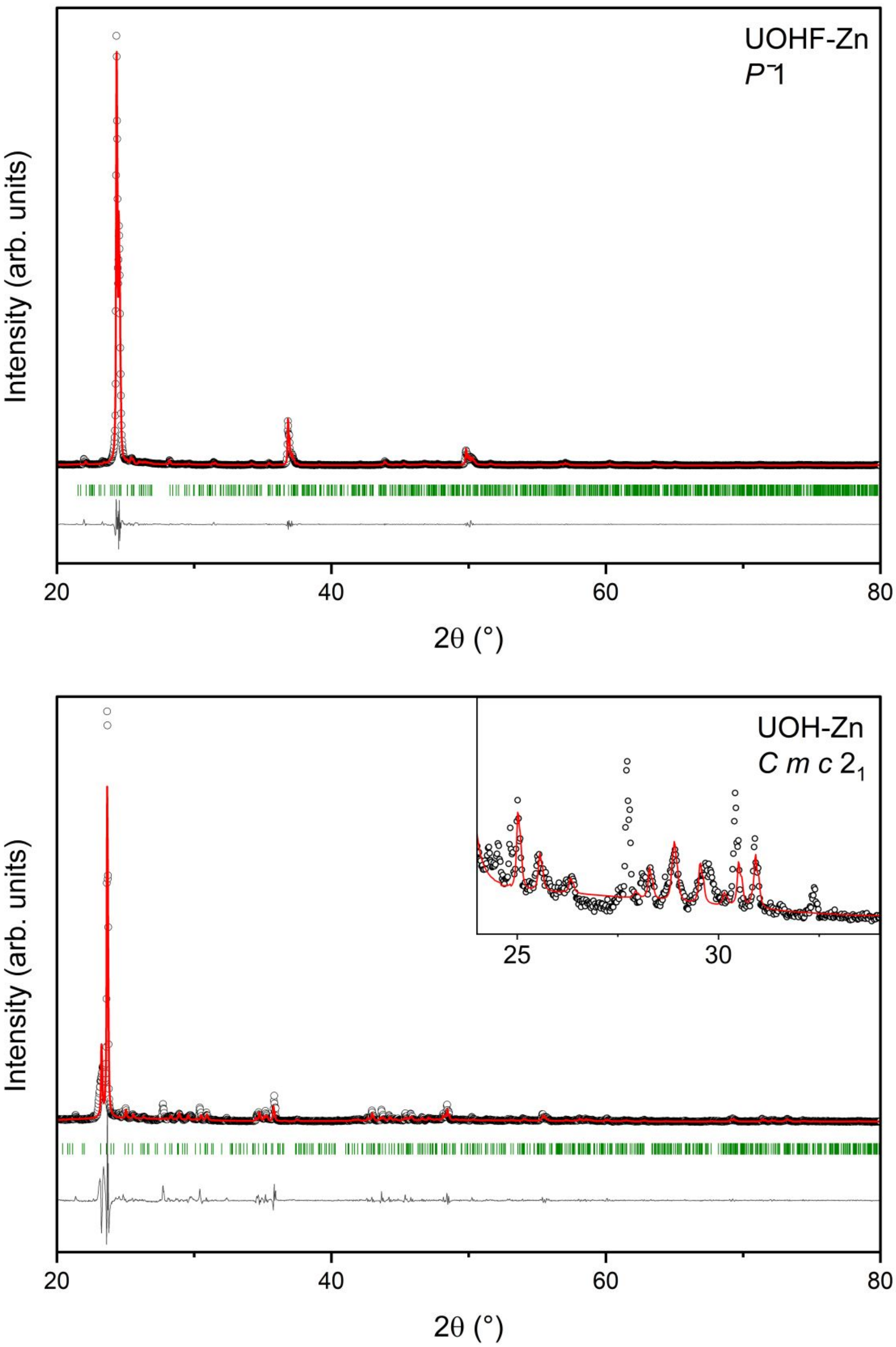

Figure S2. Powder XRD patterns of **UOHF-Zn** (top) and **UOH-Zn** (bottom) indexed with a Le Bail fitting method. Allowed hkl marks are shown in green, the collected data is shown as black circles and a fitting of the phase is shown as the red line.

Table S1. BVS calculations for **UOHF-Zn**

	**U1**		**U2**		**U3**	**U4**		**U5**		**U6**	**Zn1**		**Zn2**		
**Occ**	**1**		**1**		**1**	**1**		**1**		**1**	**0.65**		**0.35**		
**Sym.**	**1**		**1**		**1**	**1**		**2**		**1**	**1**		**1**		
**CN #**	**7**		**7**		**6**	**7**		**6**		**7**	**6**		**5**	**Σ**	
O1	1.64										0.25			**1.89**	
O2	1.73													**1.73**	
O3	0.51		1.26											**1.78**	
O4			1.54										0.35	**1.89**	
O5			0.64	0.66	0.76									**2.07**	
O6					1.48						0.41			**1.89**	
O7	0.57				1.22									**1.79**	
O8					0.73	0.66	0.68							**2.08**	
O9					0.79	0.65				0.66				**2.10**	
O10						1.51								**1.51**	
O11	0.52	0.43				1.11								**2.06**	
O12						0.48		1.15	1.15	0.45				**2.08**	
O13	0.56		0.66			0.81								**2.03**	
O14			0.49					1.18	1.18	0.45				**2.12**	
O15								0.62	0.62					**0.62**	(H_2O)
O16										1.48			0.43	**1.91**	
O17										1.44	0.42			**1.87**	
O18			0.60		0.78					0.64				**2.02**	
O19										0.80				**0.80**	(H_2O)
O20											0.45			**0.45**	(H_2O)
O21											0.46	0.19	0.58	**1.23**	(OH)
O22													0.41	**0.41**	(H_2O)
O23													0.42	**0.42**	(H_2O)
**Σ**	**5.96**		**5.87**		**5.75**	**5.91**		**5.90**		**5.91**	**2.19**		**2.20**		

Table S2. BVS calculations for **UOH-Zn**

	**U1**	**U2**	**U3**	**U4**	**Cs1**		**Cs2**		**Zn1**		
**Occ**	**1**	**1**	**1**	**1**	**0.5**		**0.5**		**0.5**		
**CN #**	**7**	**7**	**7**	**7**	**11**		**8**		**6**	**Σ**	
O1	1.65								0.27	**1.92**	
O2	1.72				0.15	0.15				**2.02**	
O3	0.50	0.46		0.41						**1.37**	(OH)
O4	0.56			0.66						**1.22**	(OH)
O5	0.47	0.41	0.48							**1.36**	(OH)
O6	0.72	0.66	0.67		0.06	0.06				**2.18**	
O7		1.56							0.38	**1.94**	
O8		1.50			0.11	0.11	0.12	0.12		**1.97**	
O9		0.55	0.41	0.51						**1.47**	
O10		0.71	0.66	0.66			0.11	0.11		**2.25**	
O11			1.62		0.14	0.14			0.24	**2.14**	
O12			1.59				0.08	0.08		**1.76**	
O13	0.35		0.45	0.47						**1.27**	(OH)
O14				1.72			0.15	0.15		**2.01**	
O15				1.65						**1.65**	
O16					0.09				0.27	**0.36**	(H_2O)
O17					0.15				0.33	**0.47**	(H_2O)
O18									0.37	**0.37**	(H_2O)
O19					0.03					**0.03**	(H_2O)
O20										**0.00**	(H_2O)
O21										**0.00**	(H_2O)
O22										**0.00**	(H_2O)
**Σ**	**5.98**	**5.85**	**5.88**	**6.07**	**1.19**		**0.94**		**1.86**		
